# Analysis of the Complete Open Reading Frame of Genotype 2b Hepatitis C Virus in Association with the Response to Peginterferon and Ribavirin Therapy

**DOI:** 10.1371/journal.pone.0024514

**Published:** 2011-09-15

**Authors:** Makoto Kadokura, Shinya Maekawa, Ryota Sueki, Mika Miura, Kazuki Komase, Hiroko Shindo, Fumitake Amemiya, Tomoyoshi Uetake, Taisuke Inoue, Minoru Sakamoto, Mina Nakagawa, Naoya Sakamoto, Mamoru Watanabe, Nobuyuki Enomoto

**Affiliations:** 1 First Department of Internal Medicine, Faculty of Medicine, University of Yamanashi, Chuo, Yamanashi, Japan; 2 Department of Gastroenterology and Hepatology, Tokyo Medical and Dental University, Bunkyo, Tokyo, Japan; Saint Louis University, United States of America

## Abstract

**Background and Aims:**

Patients infected with genotype 2b hepatitis C virus (HCV) generally can achieve favorable responses to pegylated-interferon plus ribavirin therapy (PEG-IFN/RBV). However, a proportion of patients show poorer responses and the correlation between viral sequence variation and treatment outcome remains unclear.

**Methods:**

The pretreatment complete open reading frame (ORF) sequences of genotype 2b HCV determined by direct sequencing were investigated for correlation with the final outcome in a total of 60 patients.

**Results:**

In this study group, 87.5% (14/16) of non-sustained virological response (non-SVR) patients (n = 16) were relapsers. Compared to sustained virological response (SVR) patients (n = 44), non-SVR patients were older and could not achieve prompt viral clearance after the therapy induction. Comparing each viral protein between the two groups, viral sequences were more diverse in SVR patients and that diversity was found primarily in the E1, p7, and NS5A proteins. In searching for specific viral regions associated with the final outcome, several regions in E2, p7, NS2, NS5A, and NS5B were extracted. Among these regions, part of the interferon sensitivity determining region (ISDR) was included. In these regions, amino acid substitutions were associated with the final outcome in an incremental manner, depending upon the number of substitutions.

**Conclusions:**

Viral sequences are more diverse in SVR patients than non-SVR patients receiving PEG-IFN/RBV therapy for genotype-2b HCV infection. Through systematic comparison of viral sequences, several specific regions, including part of the ISDR, were extracted as having significant correlation with the final outcome.

## Introduction

Worldwide, 180 million people are estimated to be infected with hepatitis C virus (HCV), a major cause of chronic hepatitis, liver cirrhosis, and hepatocellular carcinoma (HCC) [1]. In HCV-infected patients with chronic hepatitis, treatment with interferon (IFN)-based therapy can result in viral clearance as well as biochemical and histological improvements [2]. In this IFN-based therapy, HCV genotype is the most significant factor affecting treatment responses [3], [4].

In genotype 2b HCV infection, 80% of patients with high viral titers can achieve a sustained virological response (SVR) to the regimen of pegylated-interferon (PEG-IFN) plus ribavirin (RBV) for 24 weeks [5], [6]. This response is high considering that much lower percentages of patients infected with other genotypes can achieve SVR, especially with genotype 1 [1]. However, in other words, 20% of patients infected with genotype 2b HCV still cannot clear the virus and remain at risk of developing HCC. On the other hand, although various studies have been undertaken to clarify the factors contributing to the response to IFN-based therapy in genotype 1 infection, it remains poorly understood which patients with genotype 2b HCV infection will show unfavorable responses. Recently, the significance of IL28B single nucleotide polymorphisms (SNPs) in determining the response to PEG-IFN/RBV therapy was demonstrated in genotype 1 HCV infection [7], [8]. However, the significance of IL28B SNPs was rather weak in genotype 2 HCV infection [9].

In terms of the association between HCV sequence variation and treatment responses, previous studies have reported that amino acid variation in the NS5A-ISDR [10], NS5A-IRRDR [11], NS5B [12], PKR-eIF2 phosphorylation homology domain (PePHD) of E2 [13], and Core [14] correlate with the clinical outcome of IFN-based therapy, including PEG-IFN/RBV therapy for genotype 1b HCV infection. In the meantime, these viral sequence studies have been controversial regarding their true clinical importance, because the results of different studies were not always coincident [15], [16], [17]. On this background, recent studies trying to analyze the correlation of complete HCV open reading frame diversity, clinical characteristics, and the response to PEG-IFN/RBV therapy for genotype 1 HCV infection, in the most comprehensive approach yet attempted, have clarified that viral amino acid variation is associated with treatment responses, with consideration of racial background [18], [19]. In genotype 2 infection, however, only a few studies have investigated the association of HCV sequence variation and treatment response [20], [21] and the clinical significance has been yet established. We reported recently that variation of amino acid (aa) 110 in Core and amino acids (aa) 2258–2308 in NS5A were significantly associated with treatment outcome of the PEG-IFN/RBV therapy for genotype 2a HCV infection, through the analysis of the complete HCV ORFs in Japanese patients [22].

In this study, to assess comprehensively the influence of viral sequence variation on the response to the PEG-IFN/RBV therapy in genotype 2b HCV infection, we determined the complete pretreatment HCV ORFs from Japanese patients and investigated amino acid variation and its correlation with the response to combination therapy with PEG-IFN plus RBV.

## Methods

### Patients

A total of 77 adult Japanese patients infected with genotype 2b HCV, who received the combination therapy with PEG-IFN (PEGINTRON®, Schering-Plough, Tokyo, Japan) plus RBV (REBETOL®, Schering-Plough) between 2005 and 2009 at University of Yamanashi, Tokyo Medical and Dental University, and related institutions were first included in the study. They all fulfilled following criteria: (1) negative for hepatitis B surface antigen, (2) high viral load (≥100 KIU/ml), (3) absence of hepatocellular carcinoma, (4) no other form of hepatitis, such as primary biliary cirrhosis, autoimmune liver disease, or alcoholic liver disease, (5) free of co-infection with human immunodeficiency virus. To clearly disclose the non-SVR viral characteristics, we have considered only those patients who achieved total drug administration of 60% or more for both PEG-IFN and RBV, with the completion of the standard treatment duration. Moreover, although we excluded patients with extended therapy to make the studied population uniform, we have included non-SVR patients with extended therapy to clarify the specific characteristics of non-SVR patients, a minor population group. As a result, 17 patients were excluded for the following reasons: 1 patient received insufficient dose, 4 patients were discontinued from the therapy within 12 weeks, and 12 SVR patients received extended therapy. Finally, 60 patients were considered as eligible for the study. During the combination therapy, blood samples were obtained at least once every month before, during and after treatment and were analyzed for blood count, ALT and HCV RNA levels. Liver biopsy specimens were obtained from most of the patients. All patients gave written informed consent to the study. The study was approved by the ethics committees of University of Yamanashi, Tokyo Medical and Dental University, and related institutions. The therapy was performed according to the standard treatment protocol of PEG-IFN/RBV therapy for Japanese patients established by a hepatitis study group of the Ministry of Health, Labour, and Welfare, Japan (PEG-IFNα-2b 1.5 µg/kg body weight, once weekly subcutaneously, and RBV 600–800 mg daily per os for 24 weeks).

### Complete HCV-ORF Sequence Determination by Direct Sequencing from Pretreatment Sera

HCV RNA was extracted from pretreatment serum samples by the AGPC method using Isogen (Wako, Osaka, Japan) according to the following protocol. Briefly, 150 µl of serum were mixed with 700 µl of Isogen, and an aqueous phase was extracted with 150 µl of chloroform. RNA was precipitated with 600 µl of isopropanol and with 2 µl of Glyco Blue (Ambion, Tokyo, Japan) as a carrier. The purified RNA was washed once with ethanol and finally dissolved in 15 µl of distilled water and stored at −70°C until use.

Complementary DNA was synthesized according to the following protocol. 30 µl of the reverse transcription mixture were adjusted to contain 3 µl of the RNA solution, 300 U of Superscript II (Invitrogen, Tokyo, Japan) with an accompanied buffer according to the manufacturer's instructions, 60 units of RNase inhibitor (Promega Corp., Madison, WI), and 300 pg of random primers (Invitrogen). The mixture was incubated at 37°C for 30 min. The HCV genome was amplified with 24 partially overlapping primer (Table S6) sets, designed specifically for this study, to perform two-step nested PCR. As previously reported, a M13 forward primer (5′-TGTAAAACGACGGCCAGT-3′) and a M13 reverse primer (5′-CAGGAAACAGCTATGACC-3′) were attached to the 5′ termini of the sense and antisense second-round PCR primers, respectively, to facilitate direct sequencing. All samples were initially denatured at 95°C for 7 min., followed by 40 cycles with denaturation at 95°C for 15 seconds, annealing at 55°C for 15 seconds, and extension at 72°C for 45 seconds with BD Advantage™ 2 PCR Enzyme System (BD Biosciences Clontech, CA, USA). PCR amplicons were sequenced directly by Big Dye Terminator Version 3.1 (ABI, Tokyo, Japan) with universal M13 forward/M13 reverse primers using an ABI prism 3130 sequencer (ABI). The sequence files generated were assembled using Vector NTI software (Invitrogen) and base-calling errors were corrected following visual inspection of the chromatogram. When several peaks were observed at the same nucleotide position in the chromatogram, the highest chromatogram peak was read as the dominant nucleotide. In sequence analysis, multiple sequence alignment was performed with ClustalW, and the mean genetic distance was calculated using the p-distance algorithm in the MEGA version 4 DNA software. As a result, 60 genotype-2b HCV full open reading frame sequences were determined. In Table S1, obtained GenBank accession numbers for these sequences determined in this study are listed.

### Sliding Window Analysis

A sliding window analysis was introduced to search through HCV amino acid “regions”, rather than single amino acid positions, related to the final outcome of PEG-IFN/RBV therapy. Briefly, the total number of amino acid substitutions compared to the consensus sequence within a given amino acid length were counted at each amino acid position in each HCV sequence. The consensus sequence was generated from these 60 patients. Then the relation of substitution numbers and the final outcome was compared statistically between the SVR and non-SVR groups by Mann-Whitney's U test for each amino acid position. In this study, we changed the window length from 1 to 50 to search for those HCV regions. To visualize the result, significantly lower p-values were colored in red and non-significant p-values were colored in green using Microsoft Excel software to generate a “heat map” appearance. In the present study, p-value of 1/300 or lower was colored in the maximum red.

### Statistical Analysis

Statistical differences in the parameters, including all available patients' demographic, biochemical, hematological, and virological data such as sequence variation factors, were determined between the various groups by Mann-Whitney's U test for numerical variables and Fisher's exact probability test for categorical variables. To evaluate the optimal threshold of variations for SVR prediction, a receiver operating characteristic curve was constructed and the area under the curve as well as the sensitivity and specificity were calculated. Variables that achieved statistical significance (*p<0.05*) in univariate analysis were entered into multiple logistic regression analysis to identify significant independent factors. We also calculated the odds ratios and 95% confidence intervals. All p values of *<0.05* by the two-tailed test were considered significant.

## Results

### Characteristics of the patients studied

The SVR rate of the patients analyzed was 75.9% (44/58) with the standard therapy (two non-SVR patients received extended therapy). The baseline characteristics of the patients classified according to achievement of SVR are shown in Table 1. Rapid virological response (RVR; undetectable serum HCV RNA within 4 weeks) and early virological response (EVR; undetectable serum HCV RNA within 12 weeks) rates were significantly higher in SVR patients (*p = 0.0008* and *0.004*). In addition, patients with non-SVR were older (*p = 0.04*). Pretreatment HCV RNA titer, which is known to affect the treatment outcome in genotype 1 and 2a HCV infection, did not differ significantly between two groups. Achievement of RVR reached 42.4% when all patients were included, and this rate was high compared to achievement of RVR in patients with genotype 1b infection (∼10%) observed in University of Yamanashi (data not shown). The early virological response (EVR) rate was equally high in the SVR (97.7%) and non-SVR (68.8%) groups. Interestingly, most of the non-SVR patients (14/16, 87.5%) in genotype-2b HCV infection showed end-of-treatment response (ETR; undetectable serum HCV RNA at the end of therapy), demonstrating that the main cause of non-SVR was relapse (reappearance of hepatitis C viremia during the follow-up period after stopping therapy in patients with an ETR, n = 14), and not null response (detectable serum HCV RNA at the end of therapy, n = 2).

**Table 1:** Baseline Characteristics of Studied Patients.

Characteristic	SVR (n = 44)	non-SVR (n = 16)	*P* value
Gender (Male/Female)	26/18	9/7	NS†
Age (yrs)	56 (22–72)*	59 (30–80)	0.04‡
BMI	23.5 (16.6–30.3)	24.7 (18.5–31.7)	NS‡
ALT (IU/l)	51 (19–380)	41 (17–390)	NS‡
GGTP (IU/l)	36 (11–133)	40 (17–292)	NS‡
T.Chol (mg/dl)	169 (119–225)	178 (145–217)	NS‡
WBC (/µl)	4600 (2620–7200)	5080 (3270–8600)	NS‡
Hb (g/dl)	14.2 (11.5–17.3)	14.6 (11.8–16.4)	NS‡
Platelet (×10^4^/mm^3^)	19 (7.1–31.8)	17.8 (8–36.7)	NS‡
Fibrosis score (0–2/≥3)§	38/5	7/3	NS†
HCV RNA (KIU/ml)	2050 (100–16000)	1800 (140–6300)	NS‡
IFN dose (≥80%/60–80%)	36/8	13/3	NS†
Ribavirin dose (≥80%/60–80%)	32/12	10/6	NS†
RVR rate (%)	55.8	6.3	0.0008†
EVR rate (%)	97.7	68.8	0.004†
ETR rate (%)	100	87.5	NS†

§: SVR : n = 43, non-SVR : n = 10.

*: median (range).

†: Fisher's exact probability test.

‡: Mann-Whitney's U test.

### Phylogenetic analysis of SVR and non-SVR patients using the complete HCV amino acid sequence

**Figure 1:**
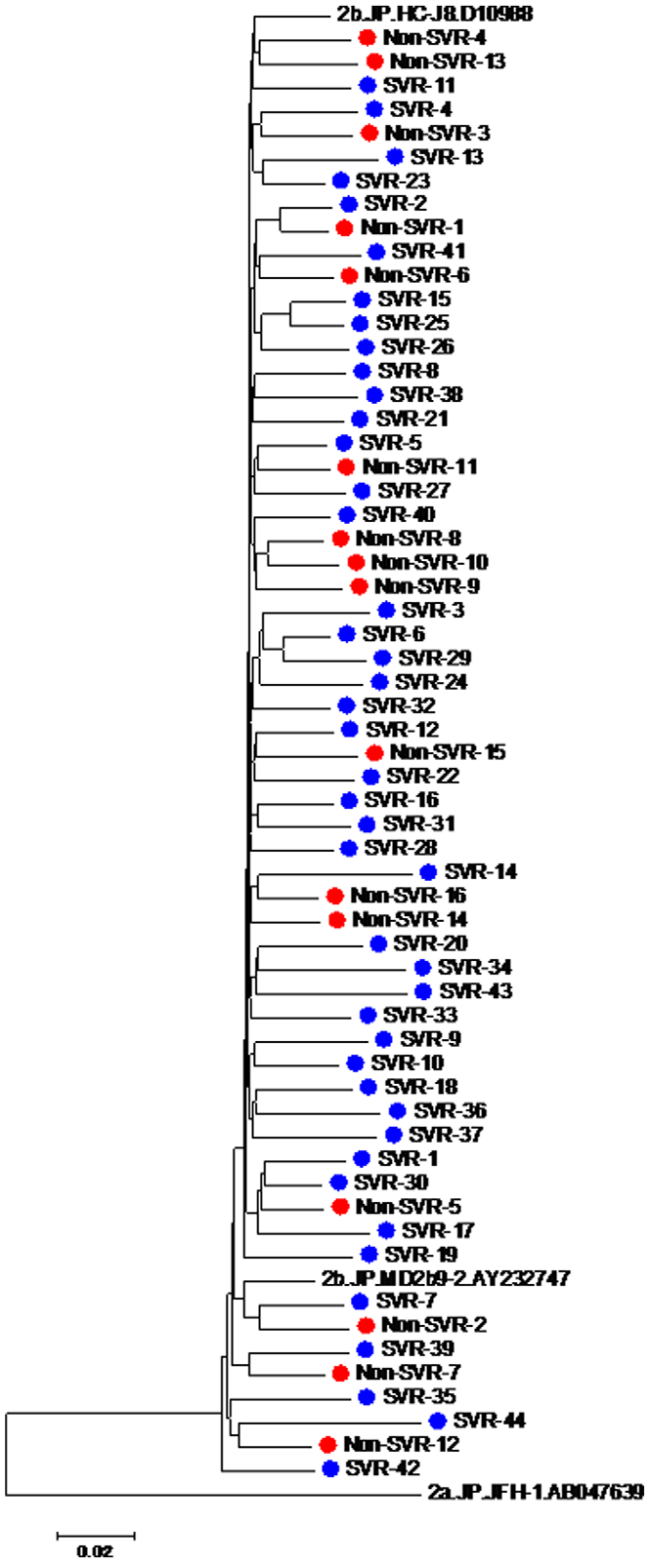
Phylogenetic analysis of the genotype-2b polyprotein sequences. In order to perform the phylogenetic analysis, we first aligned all 60 HCV complete ORF amino acid sequences obtained from the patients along with reference sequences (2b.HC-J8.D10988, 2.JP.MD2b9-2, and 2a.JP.JFH-1.AB047639), using the ClustalW program, and constructed the phylogenetic tree using the Neighbor-Joining method with MEGA version 4 software. Blue circles indicate SVR patients and red circles indicate non-SVR patients.

To determine the viral sequence characteristics in the SVR and non-SVR groups, we first aligned all 60 HCV complete ORF amino acid sequences obtained from the patients' pretreatment sera along with reference sequences (2b.HC-J8.D10988, 2.JP.MD2b9-2, and 2a.JP.JFH-1.AB047639 obtained from the Los Alamos HCV Database as representative sequences for genotype 2b and genotype 2a HCV) and constructed a phylogenetic tree (Fig. 1). As demonstrated in the tree, no evident clustering was apparent according to the difference of responses.

### Comparison of amino acid variation between the SVR and non-SVR in the complete HCV polyprotein and each HCV protein

**Figure 2:**
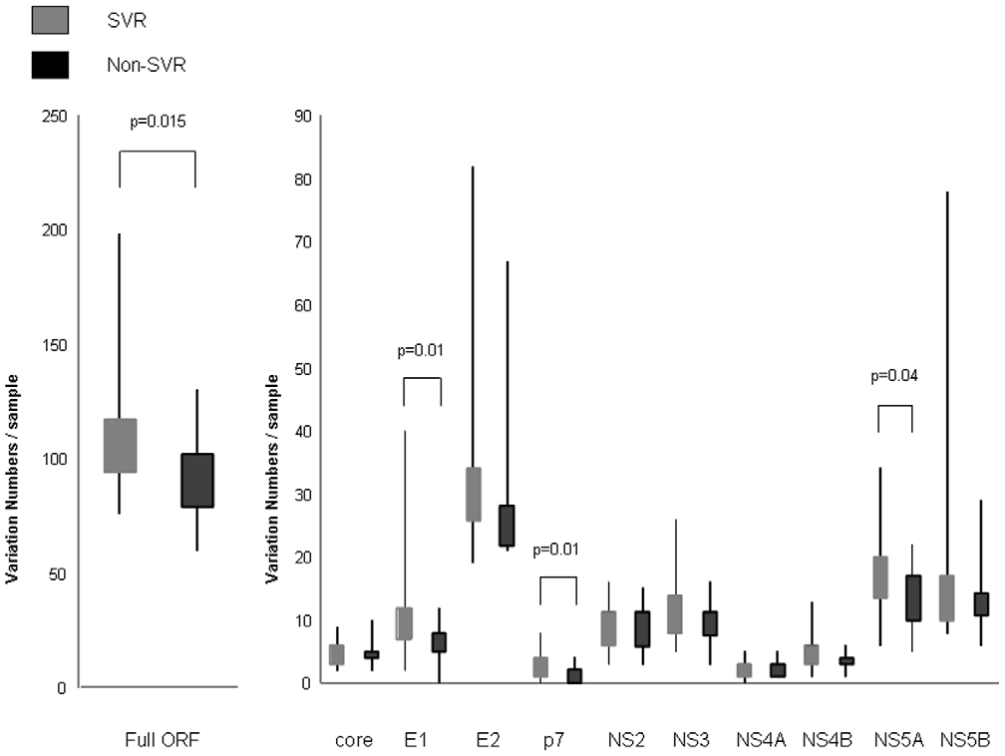
Number of amino acid substitutions per sample in the sustained virological responders (SVR) and the non-sustained virological responders (non-SVR) group. The numbers of variations, relative to a population consensus, that were unique to either SVR or non-SVR patients are shown for the complete open reading frame (ORF) (Fig. 1, left) and for each HCV protein (Fig. 1, right).

Next, we compared amino acid variations that were unique, relative to a population consensus, to either the SVR or non-SVR patients for the complete HCV polyprotein and each HCV protein. The number of amino acid variations in the sequences from the SVR patients was significantly higher than in those from the non-SVR patients, when the entire HCV polyprotein was analyzed (Fig. 2, left). These differences were especially significant in E1, p7 and NS5A (Fig. 2, right). This result demonstrated that HCV sequences from patients with SVR comprised a heterogeneous population, while HCV sequences from patients with non-SVR comprised a rather homogeneous population, indicating the existence of unique non-responsive HCV sequences in those regions in E1, p7, and NS5A.

### Comparison of HCV sequence variation between the SVR and non-SVR patients at each amino acid position

**Figure 3:**
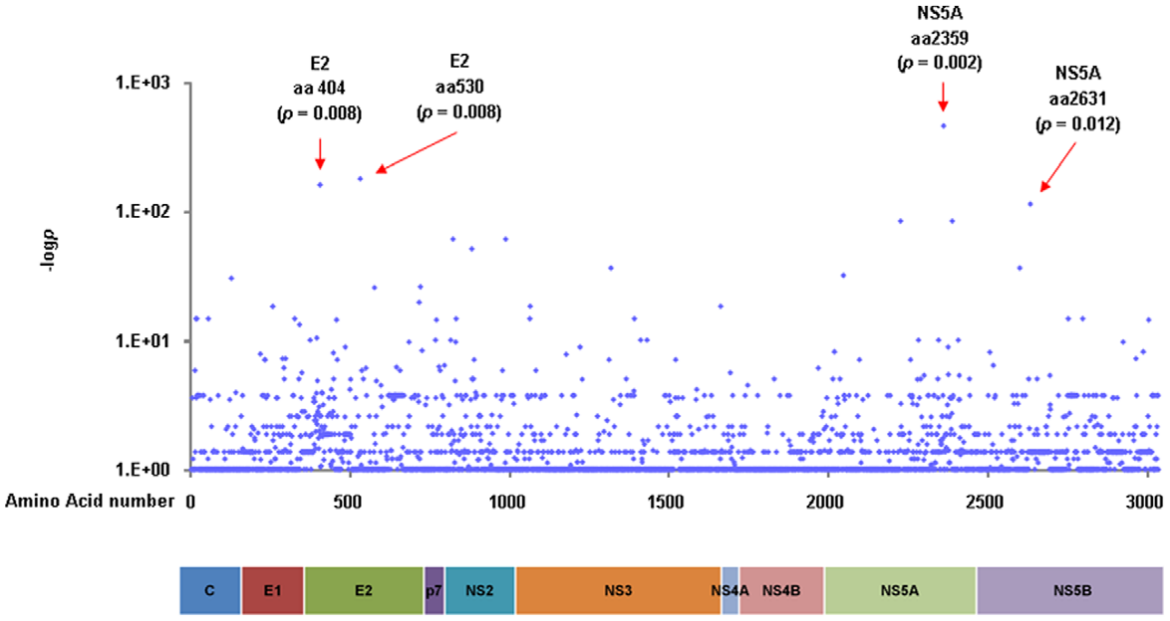
Different amino acid usage at each viral amino acid position between the sustained virological responders (SVR) and the non-sustained virological responders (non-SVR) patients. (a) Different amino acid usage at each viral amino acid position between the SVR and the non-SVR patients was analyzed by Fisher's exact probability test. The longitudinal axis shows the −logP value. (b) Sequence alignment in the Core region is demonstrated. Dashes indicate amino acids identical to the consensus sequence and substituted amino acids are shown by standard single letter codes.

Each amino acid position in the HCV ORF was compared to detect any differences between the SVR and non-SVR patients. In Fig. 3a, differences in amino acid resides at each position are shown as dots demonstrating −logP values. As shown in Table 2, four points were extracted: amino acid (aa) 404 in the E2 region (*p = 0.008*), aa 530 in the E2 region (*p = 0.008*), aa 2359 in the NS5A region (*p = 0.002*) and aa 2631 in the NS5B region (*p = 0.012*). Among them, the residue at aa 2359 in the NS5A region differed most frequently between the SVR and non-SVR patients. Amino acids 4 and 110 in the Core region, residues that have been reported to vary according to the virological responses in genotype 2a infection [22], [23], did not differ significantly in this genotype 2b HCV study. Meanwhile, amino acids 70 and 91, which have been reported to vary according to virological response to PEG-IFN/RBV therapy in genotype 1b infection, were conserved irrespective of the outcome (Fig. 3b).

**Table 2:** Variation at each Amino Acid Position and SVR rate.

	E2aa 404 non T	E2aa 530 non T	NS5Aaa 2359 N	NS5Baa 2631 non P
SVR rate	86.1%(31*/36**, p = 0.008)	87.9%(29/33, p = 0.008)	82%(41/50, p = 0.002)	94.7%(18/19, p = 0.012)

*SVR number in patients fulfilling the criteria.

**Number of patients fulfilling the criteria.

### Comparison of amino acid variation between the SVR and non-SVR patients across HCV “regions” using sliding window analysis

**Figure 4:**
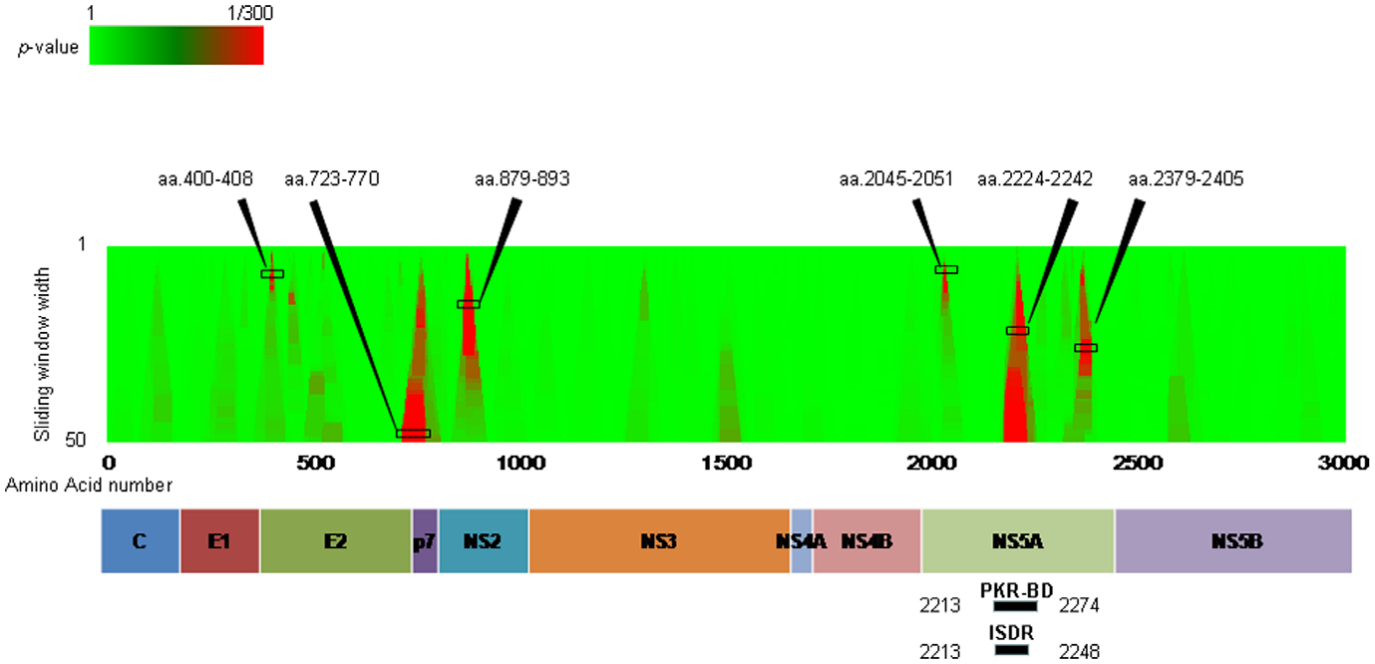
Sliding window analysis. (a) Comparison of amino acid variation between the SVR and non-SVR patients across HCV “regions” using sliding window analysis was performed. Viral regions affecting treatment outcome are shown as red areas. There are six hot areas: amino acid 400–408 and 723–770 in the E2 region, amino acid 879–893 in the NS2 region and, amino acid 2045–2051, 2224–2242 and 2379–2405 in the NS5A region. (b) Sequence alignment in the nonstructural (NS)5A around amino acids 2213 to 2274 is demonstrated. Dashes indicate amino acids identical to the consensus sequence and substituted amino acids are shown by standard single letter codes.

Fig. 4a and Table 3 shows the result of sliding window analysis. This approach was used to detect differing HCV amino acid “regions”, rather than single amino acid positions, between the SVR and the non-SVR patients. According to the result, six regions were associated with the final outcome (p-values less than 1/20): aa 400–408 in the E2 region (*p = 0.006*), aa 723–770 in the E2 and the N-terminus of p7 region (*p = 0.001*), aa 879–893 in the NS2 region (*p = 0.01*), aa 2045–2051 in the NS5A region (*p = 0.000*2), aa 2224–2242 in the NS5A region (*p = 0.001*) and aa 2379–2405 in the NS5A region (*p = 0.03*). Interestingly, aa 2224–2242 in the NS5A was located in the interferon sensitivity determining region (ISDR). Fig. 4b shows the aligned sequences of amino acids around 2213–2274 of HCV NS5A. Among these 6 regions, aa 723–770, aa 879–893, aa 2224–2242, and aa 2379–2405 were correlated with the final outcome in an incremental manner according to the number of substitutions in those regions (Table S2, S3, S4, S5). The number of substitutions in the ISDR was also correlated to the final outcome in an incremental step-up manner (data not shown).

**Table 3:** Number of Amino Acid Substitutions in each Region and SVR rate.

	E2aa 400–408mutation ≥4	E2aa 723–770mutation ≥2	NS2aa 879–893mutation ≥2	NS5Aaa 2045–2051absense of mutation	NS5AISDR(aa 2213–2248)mutation ≥1	NS5Aaa 2224–2242mutation ≥1	NS5Aaa 2379–2405mutation ≥2
SVR rate	86.5% (32*/37**)p = 0.006	100% (18/18)p = 0.001	94.7% (18/19)p = 0.01	89.7% (35/39)p = 0.0002	86.1% (31/36)p = 0.008	90.9% (30/33)p = 0.001	90.9% (20/22)p = 0.03

*SVR number in patients fulfilling the criteria.

**Number of patients fulfilling the criteria.

### Multivariate analysis to detect independent predictive factors contributing to the SVR

Next, multivariate analysis was undertaken to identify pretreatment variables correlated with the final outcome. To evaluate the optimal threshold of amino acid variations for SVR prediction in each viral region extracted, a receiver operating characteristic curve was constructed and the most optimal cut off value was determined for each region. E2 aa404–408 was excluded from the analysis because we considered that the region was unlikely to be truly associated to the outcome as it is located in the hypervariable region, the region of the highest mutation rate in the HCV genome as a result of host's immune attack. E2 aa 723–770 was excluded from the analysis because all the patients above the cut-off value in the region achieved SVR and an odds calculation was not possible. The ISDR was also excluded because NS5A aa2224–2242 was completely contained in the ISDR. In addition, variables of EVR and RVR were excluded because they were post treatment variables. The multivariate analysis revealed that only NS5A aa 2224–2242 (odds ratio 11.0, *p = 0.039*) was finally identified as the independent variable predicting the final outcome (Table 4).

**Table 4:** Multivariate Logistic Regression Analysis.

Factor	odds (95% CI)	p value
Age	0.94 (0.85–1.04)	0.20
E2 aa 530 non T	4.33 (0.48–39.3)	0.19
NS5A aa 2359 N	3.22 (0.18–57.7)	0.43
NS5B 2631 non P	5.14 (0.29–91.2)	0.26
NS2 aa 879–893 mutations ≥2	9.77 (0.52–182)	0.13
NS5A aa 2045–2051 no mutations	4.46 (0.39–50.6)	0.23
NS5A aa 2224–2242 mutations ≥1	11.0 (1.13–107)	0.04
NS5A aa 2379–2405 mutations ≥1	7.03 (0.62–79.8)	0.12

To evaluate the optimal threshold of amino acid variations for SVR prediction in each viral region extracted, a receiver operating characteristic curve was constructed and the most optimal cut off value was determined for each region.

### Biological relevance of variation in NS5A in this study group

**Figure 5:**
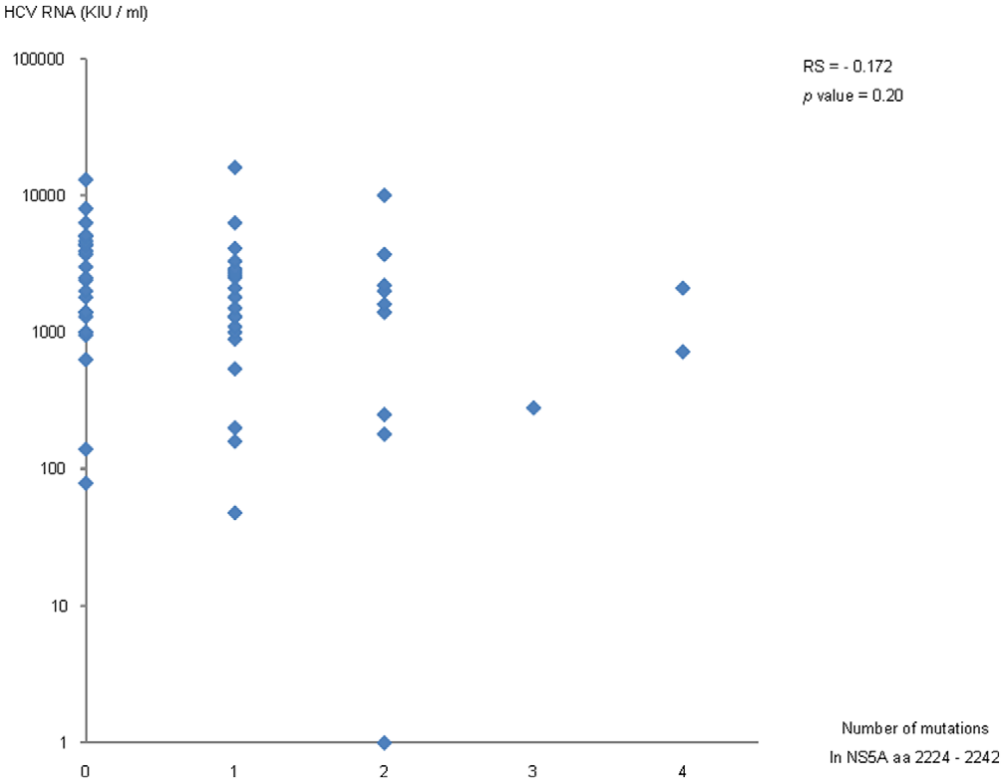
Correlation between pretreatment HCV RNA levels and the number of substitutions in the NS5A region aa 2224 to 2242. Spearman's correlation coefficient by rank test is demonstrated.

Because NS5A aa 2224–2242 is located within the ISDR, for which the amino acid substitution numbers have been reported to be correlated with the HCV RNA titer in genotype 1 and 2a HCV infection [13], we analyzed the relationship between amino acid variations in that region and pretreatment HCV RNA titers. Contrary to our expectation, no evident relationship was found between variations in the NS5A region aa 2224–2242 and HCV RNA titer (Fig. 5). On the other hand, as shown in Table 5, although the initial viral responses (RVR or EVR) did not show evident association with the amino acid variations in the region, treatment relapse was significantly correlated with the amino acid variations in the region. In addition to NS5A aa 2224–2242, there was no evident relationship between HCV RNA level and variations in the other regions found in this study (data not shown).

**Table 5:** Baseline Characteristics of patients with NS5A aa 2224–2242 variations none or 1≤.

Characteristic	Variation 1≤ (n = 33)	No variation (n = 27)	*P* value
Gender (Male/Female)	17/16	18/9	NS†
Age (yrs)	57 (29–72)*	57 (22–80)	NS‡
ALT (IU/l)	72 (19–380)	47 (17–390)	NS‡
Platelet (×10^4^/mm^3^)	19.3 (7.1–31.8)	17.5 (10.4–36.7)	NS‡
Fibrosis score (0–2/≥3)§	26/5	19/3	NS†
HCV RNA (KIU/ml)	1600 (100–16000)	2450 (140–13000)	NS‡
IFN dose (≥80%/60–80%)	26/7	23/4	NS†
Ribavirin dose (≥80%/60–80%)	24/9	19/8	NS†
RVR rate (%)	53.1	29.6	NS†
EVR rate (%)	96.9	81.5	NS†
SVR rate (%)	90.9	51.9	0.001†
Replapse rate (%)	40.7	9.1	0.006†

§ : 1≤ : n = 31, 0 : n = 22.

* : median (range).

† : Fisher's exact probability test.

‡ : Mann-Whitney's U test.

## Discussion

In this study, we showed that genotype 2b HCV sequences from Japanese patients who achieved SVR were more diverse than the sequences from patients with non-SVR. The result that SVR patients were more diverse in their HCV sequences than non-SVR patients is in accordance with previous studies of genotype 1 HCV infection, although the diverse viral genes varied according to genotype [18], [19]. We found that these diversities were primarily found in E1, p7 and NS5A.

In systemic searching for single amino acid positions or consecutive amino acid regions in the HCV ORF associated with the treatment outcome, several regions were extracted in E2, p7, NS2, NS5A and NS5B. Among those identified regions, E2 aa 723–770, NS2 aa 879–893, NS5A aa2224–2242, and NS5A aa2379–2405 were correlated with the final outcome in an incremental manner according to the number of amino acid substitutions. Specifically, the sequences of those regions in non-SVR patients were almost homogeneous, while the sequences of the region in SVR patients were significantly diverse and multiple amino acid substitutions were found compared to the consensus sequence. Interestingly, among those regions, aa 2224–2242 was completely included in the ISDR, in which the number of amino acid substitutions is known to show significant correlation with the treatment response to IFN-based therapy in genotype 1b, and also in genotype 2 [21], [24].

In recent studies of genotype 1b infection, amino acid variation of residues 70 and 91 in the Core were reported to be associated with the treatment response to IFN-based therapy. The correlation of amino acid variation in the Core (residues 4 and 110) with the response to PEG-IFN/RBV therapy was also identified in genotype 2a infection [22], [23]. In genotype 2b infection, however, we could not find such associations between amino acid variation in the core region and the response to PEG-IFN/RBV therapy (Fig. 3b). Amino acid residues of aa 70 and 91 were conserved irrespective of differences in the PEG-IFN/RBV responses. On the other hand, although amino acid variations were also sometimes found at residues 4 and 110 in genotype 2b HCV, their frequency was low, and no evident association between the variation and the treatment response was found. Although the reason of the lack of association between the Core and the PEG-IFN/RBV treatment response in genotype-2b HCV infection is unknown, it suggests that a different mechanism affecting the treatment response might exist, depending on genotype-specific viral features.

In genotype 1 HCV, variations within the PKR-binding region of NS5A, including those within the ISDR, were reported to disrupt the NS5A-PKR interaction, possibly rendering HCV sensitive to the antiviral effects of interferon [25]. Clinically, the number of substitutions within the ISDR has been reported to correlate with the serum HCV RNA level in genotype 1 and 2a infections [13]. In addition, a recent study reported that mutations in the ISDR also show the correlation with the relapse in the PEG-IFN/RBV therapy in genotype 1b infection [26]. Because NS5A aa2224–2242, part of ISDR, was extracted as one of those regions related to the treatment response in genotype 2b infection, we undertook further analysis to investigate the correlation between amino acid variation numbers and serum HCV RNA level. Though the reason is unknown, we could not find evidence of a relationship between variation in the NS5A aa 2224–2242 and HCV RNA titer in genotype 2b infection, unlike genotypes 1 and 2a. Of note, a high SVR rate in genotype1 and genotype 2a infection is known to be closely correlated with a low HCV RNA level and multiple substitutions in ISDR. However, in genotype 2b infection in our study, there was no significant difference in the HCV RNA level between SVR and non-SVR patients, as shown in Table 1. Previously, the role of the ISDR in the contribution to SVR in genotype 1 and 2a has been discussed in detail in the context of serum HCV RNA level, and multiple substitutions in the ISDR are related to a low HCV RNA level and high SVR rate. However, it is not known which of these two factors is directly associated with viral clearance. Consideration of this three-sided relationship of ISDR, HCV RNA level and SVR rate in genotype-2b infection leads to the suggestion that amino acid variation in ISDR to be more direct contributor for SVR.

In spite of these findings, there were still limitations in our study. First, because genotype 2b infection only accounts for 10% of all HCV infection in Japan, the number of studied patients was rather small, especially non-SVR patients. In addition, because genotype 2b HCV contains as many as 3033 amino acids, it is possible that incorrect amino acids or regions were judged as significant in the complete HCV ORF comparison study as a result of type I errors. Therefore, if more patients were available for the analysis, the statistical power detecting the meaningful differences would be greater. Secondly, we could not include the IL28B SNP analysis in this study. If we could have combined the information of IL28B SNPs with the full HCV ORF information, a more comprehensive analysis would have been achieved.

In conclusion, we have shown that viral sequences were more diverse in SVR patients infected with genotype 2b HCV. Through systematic comparison between SVR and non-SVR patients, we have also shown that several localized regions were extracted as hot spots whose amino acid substitutions were closely related to the final outcome by affecting the relapse rate in the PEG-IFN/RBV therapy.

## Supporting Information

Table S1 **GenBank Accession Numbers.** Obtained GenBank accession numbers for 60 genotype-2b HCV full open reading frame sequences are listed.
(DOC)

Table S2 **Substitutions in NS5A aa 2224–2242 Amino Acid Regions and SVR rate.** SVR rate increased with the number of substitutions in this region.
(DOC)

Table S3 **Substitutions in NS5A aa 2379–2405 Amino Acid Regions and SVR rate.** SVR rate increased with the number of substitutions in this region.
(DOC)

Table S4 **Substitutions in NS2 aa 879–893 Amino Acid Regions and SVR rate.** SVR rate increased with the number of substitutions in this region.
(DOC)

Table S5 **Substitutions in E2 aa 723–770 Amino Acid Regions and SVR rate.** SVR rate increased with the number of substitutions in this region.
(DOC)

Table S6 **PCR Primer List.** Primers designed to perform two-step nested PCR for this study are listed. Dominant genotype-2b HCV full open reading frame sequences was determined by the 24 partially overlapping amplicons amplified by these primers.
(XLS)
