# Supplementary material for: Analysis of the Complete Open Reading Frame of Genotype 2b Hepatitis C Virus in Association with the Response to Peginterferon and Ribavirin Therapy
Source: PLoS One. 2011 Sep 15;6(9):e24514. doi: 10.1371/journal.pone.0024514 (PMC3174186; doi:10.1371/journal.pone.0024514)
Supplement: Table S1 — GenBank Accession Numbers. Obtained GenBank accession numbers for 60 genotype-2b HCV full open reading frame sequences are listed. (DOC) [file pone.0024514.s001.doc]

Table S1. Accession numbers

| **SVR (N = 44)** | **non-SVR (N = 16)** |
| --- | --- |
| **AB661373** | AB661414 |
| **AB661374** | AB661415 |
| **AB661375** | AB661416 |
| **AB661376** | AB661417 |
| **AB661377** | AB661418 |
| **AB661378** | AB661419 |
| **AB661379** | AB661420 |
| **AB661380** | AB661421 |
| **AB661381** | AB661422 |
| **AB661382** | AB661423 |
| **AB661383** | AB661424 |
| **AB661384** | AB661425 |
| **AB661385** | AB661426 |
| **AB661386** | AB661427 |
| **AB661387** | AB661428 |
| **AB661388** | AB661429 |
| **AB661389** |  |
| **AB661390** |  |
| **AB661391** |  |
| **AB661392** |  |
| **AB661393** |  |
| **AB661394** |  |
| **AB661395** |  |
| **AB661396** |  |
| **AB661397** |  |
| **AB661398** |  |
| **AB661399** |  |
| **AB661400** |  |
| **AB661401** |  |
| **AB661402** |  |
| **AB661403** |  |
| **AB661404** |  |
| **AB661405** |  |
| **AB661406** |  |
| **AB661407** |  |
| **AB661408** |  |
| **AB661409** |  |
| **AB661410** |  |
| **AB661411** |  |
| **AB661412** |  |
| **AB661413** |  |
| **AB661430** |  |
| **AB661431** |  |
| **AB661432** |  |
