# Supplementary material for: Analysis of the Complete Open Reading Frame of Genotype 2b Hepatitis C Virus in Association with the Response to Peginterferon and Ribavirin Therapy
Source: PLoS One. 2011 Sep 15;6(9):e24514. doi: 10.1371/journal.pone.0024514 (PMC3174186; doi:10.1371/journal.pone.0024514)
Supplement: Table S2 — Substitutions in NS5A aa 2224–2242 Amino Acid Regions and SVR rate. SVR rate increased with the number of substitutions in this region. (DOC) [file pone.0024514.s002.doc]

Table S2. Substitutions in NS5A aa 2224-2242 Amino Acid Regions and SVR rate

| Substitution number | 0 | 1 | 2 | 3≤ |
| --- | --- | --- | --- | --- |
| SVR patients | 13 | 19 | 9 | 3 |
| Non-SVR patients | 14 | 2 | 1 | 0 |
| SVR rate | 48% (13/27) | 90% (19/21) | 90% (9/10) | 100% (3/3) |
