# Supplementary material for: Analysis of the Complete Open Reading Frame of Genotype 2b Hepatitis C Virus in Association with the Response to Peginterferon and Ribavirin Therapy
Source: PLoS One. 2011 Sep 15;6(9):e24514. doi: 10.1371/journal.pone.0024514 (PMC3174186; doi:10.1371/journal.pone.0024514)
Supplement: Table S3 — Substitutions in NS5A aa 2379–2405 Amino Acid Regions and SVR rate. SVR rate increased with the number of substitutions in this region. (DOC) [file pone.0024514.s003.doc]

Table S3. Substitutions in NS5A aa 2379-2405 Amino Acid Regions and SVR rate

| Substitution number | 0 | 1 | 2 | 3≤ |
| --- | --- | --- | --- | --- |
| SVR patients | 7 | 17 | 13 | 7 |
| Non-SVR patients | 10 | 4 | 2 | 0 |
| SVR rate | 41% (13/27) | 81% (17/21) | 87% (13/15) | 100% (7/7) |
