# Supplementary material for: Analysis of the Complete Open Reading Frame of Genotype 2b Hepatitis C Virus in Association with the Response to Peginterferon and Ribavirin Therapy
Source: PLoS One. 2011 Sep 15;6(9):e24514. doi: 10.1371/journal.pone.0024514 (PMC3174186; doi:10.1371/journal.pone.0024514)
Supplement: Table S4 — Substitutions in NS2 aa 879–893 Amino Acid Regions and SVR rate. SVR rate increased with the number of substitutions in this region. (DOC) [file pone.0024514.s004.doc]

Table S4. Substitutions in NS2 aa 879-893 Amino Acid Regions and SVR rate

| Substitution number | 0 | 1 | 2 |  |
| --- | --- | --- | --- | --- |
| SVR patients | 5 | 21 | 18 |  |
| Non-SVR patients | 12 | 3 | 1 |  |
| SVR rate | 29% (5/17) | 88% (21/24) | 95% (18/19) |  |
