# Supplementary material for: Analysis of the Complete Open Reading Frame of Genotype 2b Hepatitis C Virus in Association with the Response to Peginterferon and Ribavirin Therapy
Source: PLoS One. 2011 Sep 15;6(9):e24514. doi: 10.1371/journal.pone.0024514 (PMC3174186; doi:10.1371/journal.pone.0024514)
Supplement: Table S5 — Substitutions in E2 aa 723–770 Amino Acid Regions and SVR rate. SVR rate increased with the number of substitutions in this region. (DOC) [file pone.0024514.s005.doc]

Table S5. Substitutions in E2 aa 723-770 Amino Acid Regions and SVR rate

| Substitution number | 0 | 1 | 2 |  |
| --- | --- | --- | --- | --- |
| SVR patients | 11 | 15 | 18 |  |
| Non-SVR patients | 10 | 6 | 0 |  |
| SVR rate | 52% (11/21) | 71% (15/21) | 100% (18/18) |  |
